# Minimally invasive mitral valve repair revisited: Respect or Resect? Amidst competing risks

**DOI:** 10.1186/s13019-026-04350-z

**Published:** 2026-06-29

**Authors:** R. W. Brooks, P. Biaggi, O. Gaemperli, M. Y. Emmert, S. Jacobs, J. Gruenenfelder, T. Holubec, D. Reser

**Affiliations:** 1 University of Zurich, Rämistrasse 71, Zurich, 8006 Switzerland; 2 Department of Cardiac Surgery and Cardiology, Heart Clinic, Hirslanden Hospital, Zurich, Switzerland; 3 Department of Cardiothoracic and Vascular Surgery, Deutsches Herzzentrum der Charité (DHZC), Berlin, Germany; 4 Charité – Universitätsmedizin Berlin, Berlin, Germany; 5 Department of Cardiac Surgery, Cardiovascular Center, Klinikum Nürnberg, Paracelsus Medical University, Nuremberg, Germany

**Keywords:** Mitral valve repair, Degenerative mitral regurgitation, Minimally invasive cardiac surgery, Leaflet resection, Neochordae

## Abstract

**Background:**

Mitral valve repair (MVr) remains the preferred surgical treatment for degenerative mitral regurgitation (DMR), offering superior long-term outcomes compared with mitral valve replacement (MVR). However, the optimal surgical strategy—leaflet preservation with or without neochordal implantation (‘Respect’) versus leaflet resection (‘Resect’), or a combination thereof (‘Both’)—remains a matter of debate. Our aim was to compare the longer-term outcomes of these techniques in minimally invasive mitral valve surgery, in a real-world cohort encompassing anatomically complex valve pathology.

**Methods:**

In this single-center, retrospective cohort study, 447 consecutive patients who underwent isolated MVr via right lateral mini-thoracotomy between 2006 and 2014 were included and analyzed. Patients were stratified according to the surgical repair technique (‘Respect’, ‘Resect’, or ‘Both’). Primary endpoints were valve performance measures: freedom from mitral valve–related reoperation and mitral regurgitation severity during follow-up. Secondary endpoints were all-cause mortality and major adverse cardiac and cerebrovascular events (MACCE). Competing risk regression (Fine–Gray) was applied to account for death as a competing event.

**Results:**

Of the total cohort, 293 patients (65.5%) underwent MVr using the ‘Respect’ technique, 109 (24.4%) underwent leaflet resection (‘Resect’), and 45 (10.1%) received a combined approach (‘Both’). In-hospital mortality was 0.7%, and cerebrovascular events occurred in 0.9% of patients. At a mean follow-up of 5 years, durable mitral valve competence was present in 88.4% of patients (MR grade ≤ I). Five-year freedom from mitral valve–related reoperation was 93.3%, with no significant differences between techniques (*P* = 0.647). Five-year survival was 94.0%, and 5-year freedom from MACCE was 96.8%, with no statistically significant differences between techniques. Competing risk analysis (death as competing event) confirmed the absence of significant intergroup differences.

**Conclusion:**

This study demonstrates that all three techniques yield equally favorable longer-term outcomes in a minimally invasive setting, extending even to anatomically complex degenerative pathologies such as Barlow’s disease, bileaflet involvement, and anterior leaflet prolapse. A morphology-guided, individualized surgical approach emerges as a reliable and durable standard for the management of DMR.

## Background

Mitral regurgitation (MR) is among the most prevalent valvular heart diseases in Western populations and represents an increasing public health burden in aging societies [1, 2]. Degenerative mitral regurgitation (DMR) accounts for the majority of surgical referrals, and early mitral valve repair (MVr) is consistently associated with improved long-term survival, superior preservation of left ventricular (LV) function, and a reduced incidence of heart-failure–related events compared with delayed intervention or valve replacement [3–7]. Accordingly, current ESC/EACTS guidelines recommend MVr as the preferred treatment for severe primary MR in suitable patients, emphasizing early referral to experienced Heart Valve Centers and morphology-tailored repair strategies [6]. The conceptual foundations of contemporary MVr were laid by Carpentier, whose “French correction” established a systematic, resection-based philosophy aimed at restoring leaflet geometry through targeted excision and annuloplasty [8]. Subsequent leaflet-preserving approaches, notably chordal replacement techniques popularized by David, sought to maintain subvalvular continuity and native leaflet dynamics, addressing concerns related to leaflet mobility and repair-induced stress [9–12]. In clinical practice, the dichotomy between ‘Respect’ and ‘Resect’ is often artificial, particularly in advanced degenerative morphologies, where hybrid, anatomy-driven strategies combining neochordal implantation and selective resection are frequently required [13, 14].

Minimally invasive mitral valve surgery (MIMVS) has further expanded the reparative spectrum, offering outcomes comparable to sternotomy while reducing surgical trauma and facilitating recovery when performed by experienced teams [15–17]. However, comparative data on different repair strategies in the minimally invasive setting—especially in anatomically complex degenerative disease—remain limited, as most prior studies have focused on isolated posterior leaflet prolapse [18, 19].

This study aimed to assess longer-term outcomes of minimally invasive mitral valve repair (MIMVr) for DMR, comparing ‘Respect’, ‘Resect’, and hybrid strategies in a real-world cohort with particular focus on valve performance (freedom from reoperation and MR severity during follow-up), survival, and major adverse cardiovascular and cerebral events (MACCE), encompassing anatomically complex degenerative lesions.

## Methods

### Study design and patient selection

This retrospective, single-center observational cohort study used prospectively scheduled clinical and echocardiographic follow-up and was conducted at a tertiary heart center specialized in MIMVS. All adult patients undergoing primary mitral valve repair (MVr) for DMR via right lateral mini-thoracotomy between January 1, 2006, and December 31, 2014, were eligible for inclusion. The study period concluded in 2014, coinciding with a change in departmental leadership, after which MIMVS was no longer maintained as the standard institutional approach. Accordingly, this cohort represents the historical period during which MIMVS constituted the standard practice at our center. The study was approved by the Cantonal Ethics Committee Zurich (KEK-No. PB_2018 − 00249) and conducted in accordance with the Declaration of Helsinki (2013 revision). Written informed consent for study participation was obtained from all patients; follow-up assessments were conducted as part of routine clinical care.

Exclusion criteria comprised patients requiring combined procedures via sternotomy, including severe coronary artery disease with or without acute or chronic ischemic MR; concomitant tricuspid valve surgery, redo mitral valve surgery, or planned minimally invasive mitral valve replacement, as well as grade III–IV aortic atherosclerosis, severe mitral annular calcification, or suspected right pleural adhesions due to prior thoracic surgery or irradiation.

By design, the analytic cohort was confined to patients in whom MVr was successfully achieved via right mini-thoracotomy and constituted the definitive surgical treatment. Patients in whom MVR was ultimately performed—either as the primary strategy or following intraoperative conversion from an initially intended repair—were not included in the primary outcome analyses, as they no longer fulfilled the definition of a reconstructed native valve and therefore did not qualify for inclusion in the MVr cohort. These patients are described separately in the Results section to maintain methodological clarity and prevent conflation of repair- and replacement-related outcomes.

### Endpoints and outcome definitions

The primary endpoints of the study were freedom from mitral valve–related reoperation and mitral valve competence, assessed as MR grade during follow-up. The secondary endpoints were all-cause mortality and the incidence of major adverse cardiac and cerebrovascular events (MACCE), defined as a composite of non-fatal stroke or transient ischemic attack and myocardial infarction.

Additional perioperative and functional outcomes included NYHA functional class and the occurrence of early postoperative complications, such as stroke, new-onset atrial fibrillation, permanent pacemaker implantation, surgical re-exploration, and clinically significant infections.

All outcomes were systematically assessed during the index hospitalization and updated through prospective follow-up with treating physicians and cardiologists.

### Surgical techniques

All surgical procedures were performed by experienced MIMVr surgeons using a standardized right mini-thoracotomy approach, as previously described [15, 20, 21]. Annular stabilization was routinely achieved using a complete annuloplasty ring (Physio II^®^, Edwards Lifesciences, Irvine, CA, USA), implanted in the vast majority of cases.

In cases of excess leaflet tissue—particularly involving the P2 segment—a triangular resection was performed to reduce redundancy, avoid tension, and optimize posterior leaflet mobility and coaptation. When prolapse was attributable to chordal elongation or rupture without significant tissue excess, neochordae were implanted using single Gore-Tex^®^ sutures or Gore-Tex^®^ loops (Gore, Newark, DE, USA), typically two to four per segment, depending on the extent and location of the prolapse. Neochordae were placed on the prolapsing segments of the posterior and/or anterior leaflet as required.

If the leaflet anatomy did not necessitate immediate resection or chordal replacement, the annuloplasty ring was implanted first, followed by intraoperative reassessment to determine the need for additional repair techniques. Supplementary procedures—including sliding plasty of the P2 segment, single-suture commissuroplasty, indentation closure, and the Alfieri edge-to-edge stitch—were selectively employed, either as standalone interventions or in combination with the primary repair strategy.

When indicated, concomitant procedures such as closure of a patent foramen ovale (PFO), left atrial appendage (LAA) occlusion, or left atrial cryoablation were performed.

### Baseline assessment, echocardiographic evaluation and follow-up protocol

Baseline data were prospectively collected for all patients and included demographic variables (e.g., age, sex, body mass index), clinical status (e.g., NYHA functional class, presence of atrial fibrillation, comorbidities such as diabetes mellitus, hypertension, or chronic obstructive pulmonary disease (COPD)), prior cardiovascular events and interventions, as well as calculated surgical risk according to EuroSCORE II. Baseline transesophageal echocardiography (TEE) was performed in all cases prior to hospital admission and intraoperatively to assess MR severity, left ventricular ejection fraction (LVEF), pulmonary artery systolic pressure, annular or leaflet calcification, and specific valve pathologies including segmental prolapse patterns, bileaflet prolapse, Barlow’s disease, commissural involvement, leaflet restriction, or flail, as well as to evaluate the immediate result of surgery.

Postoperative evaluation included a standardized transthoracic echocardiographic study to assess residual MR. MR severity was classified using a four-grade ordinal scale: grade 0 (none), grade I (mild), grade II (moderate), and grade III (severe).

Structured clinical and echocardiographic follow-up was scheduled at 3 months, 1 year, and annually thereafter. Follow-up assessments included evaluation of MR recurrence, NYHA functional class, arrhythmia burden, left ventricular function, and adverse events such as reoperation or hospitalization. Follow-up data were primarily obtained from referring cardiologists. In cases of missing data, follow-up was supplemented by direct contact with patients, treating physicians, or caregivers via telephone or email.

### Statistical analysis

All statistical analyses were performed using SPSS, version 29.0 (IBM Corp., Armonk, NY, USA) and R, version 4.2.1 (R Foundation for Statistical Computing, Vienna, Austria). Categorical variables were summarized as absolute and relative frequencies. Continuous variables were reported as mean values with standard deviation (SD) or as medians with interquartile range (IQR), depending on data distribution. The normality of continuous variables was assessed using histograms and the Shapiro–Wilk test. All analyses were conducted on a per-patient basis.

For group comparisons, categorical variables were analyzed using the Chi-square test or Fisher’s exact test, as appropriate. For continuous variables, normally distributed data were compared using the independent samples t-test, and non-normally distributed data using the Mann–Whitney U test. When comparing more than two groups, analysis of variance (ANOVA) was employed for normally distributed data, and the Kruskal–Wallis test for non-normally distributed variables.

Time-to-event data were analyzed using the Kaplan–Meier method to estimate overall survival, freedom from mitral valve–related reoperation, and freedom from MACCE. Differences between surgical strategies were compared using the log-rank test. Patients lost to follow-up were censored at the time of their last documented contact.

To account for competing events—specifically death precluding reoperation or MACCE—we used Fine–Gray subdistribution hazard models (R, cmprsk package) to estimate cumulative incidence functions. Separate multivariable models were constructed for reoperation and for MACCE. Subdistribution hazard ratios (sHRs) with 95% confidence intervals (CIs) and P-values are reported. The surgical repair strategy (‘Respect’, ‘Resect’, or ‘Both’) was included as a categorical covariate in all Fine–Gray models.

Covariates included in the multivariable models were selected based on established clinical relevance and the completeness of available data, and encompassed patient age at the time of surgery, body mass index (BMI), EuroSCORE II, left ventricular ejection fraction (LVEF), atrial fibrillation (AF), chronic obstructive pulmonary disease (COPD), diabetes mellitus, cardiopulmonary bypass (CPB) time, and aortic cross-clamp (ACC) time. Morphological variables (posterior mitral leaflet prolapse, Barlow’s disease) were evaluated in univariable analyses but were not included in the final multivariable models due to collinearity with the primary surgical strategy and to maintain model parsimony.

All statistical tests were two-sided, and a *P* ≤ 0.05 was considered statistically significant. Variables with zero events for the endpoint were not entered into the multivariable Fine–Gray models to avoid model instability; where applicable, these are denoted as NE (not estimable) in the tables and footnotes. No additional sensitivity analyses for differential follow-up were performed, given the high completeness of follow-up and the low frequency of MACCE and reoperation events.

## Results

### Patient characteristics

Between January 2006 and December 2014, 550 consecutive patients undergoing isolated minimally invasive mitral valve surgery were screened for inclusion. Of these, 103 patients were excluded due to concomitant procedures (*n* = 19), prior cardiac surgery (*n* = 10), mitral valve replacement (*n* = 62; 47 planned, 15 after failed repair), isolated mitral stenosis without regurgitation (*n* = 1), cardiac tumor (*n* = 1), or withdrawal of consent (*n* = 10).

Among the 62 excluded patients who ultimately underwent MVR—either minimally invasively or after conversion to median sternotomy (*n* = 9) due to technical or anatomical constraints—21 (33.9%) received a mechanical and 41 (66.1%) a bioprosthetic valve. Of the nine conversions to sternotomy, seven originated from an initially planned MVr (failed-repair pathway: ‘Respect’ *n* = 5, ‘Resect’ *n* = 1, ‘Both’ *n* = 1), whereas two represented primary MVR cases. Overall, MVR was carried out as the primary strategy in 47 patients (75.8%), while in 15 patients (24.2%) replacement was performed intraoperatively after an initially planned mitral valve repair was deemed insufficient due to persistent regurgitation. Within this failed-repair subgroup (*n* = 15), the intended strategy was predominantly chordal-based (‘Respect’, *n* = 13), with single cases of ‘Resect’ (*n* = 1) and ‘Both’ (*n* = 1). Age and sex distributions were comparable to the overall MVR population, with no indication of demographic imbalance.

**Fig. 1:**
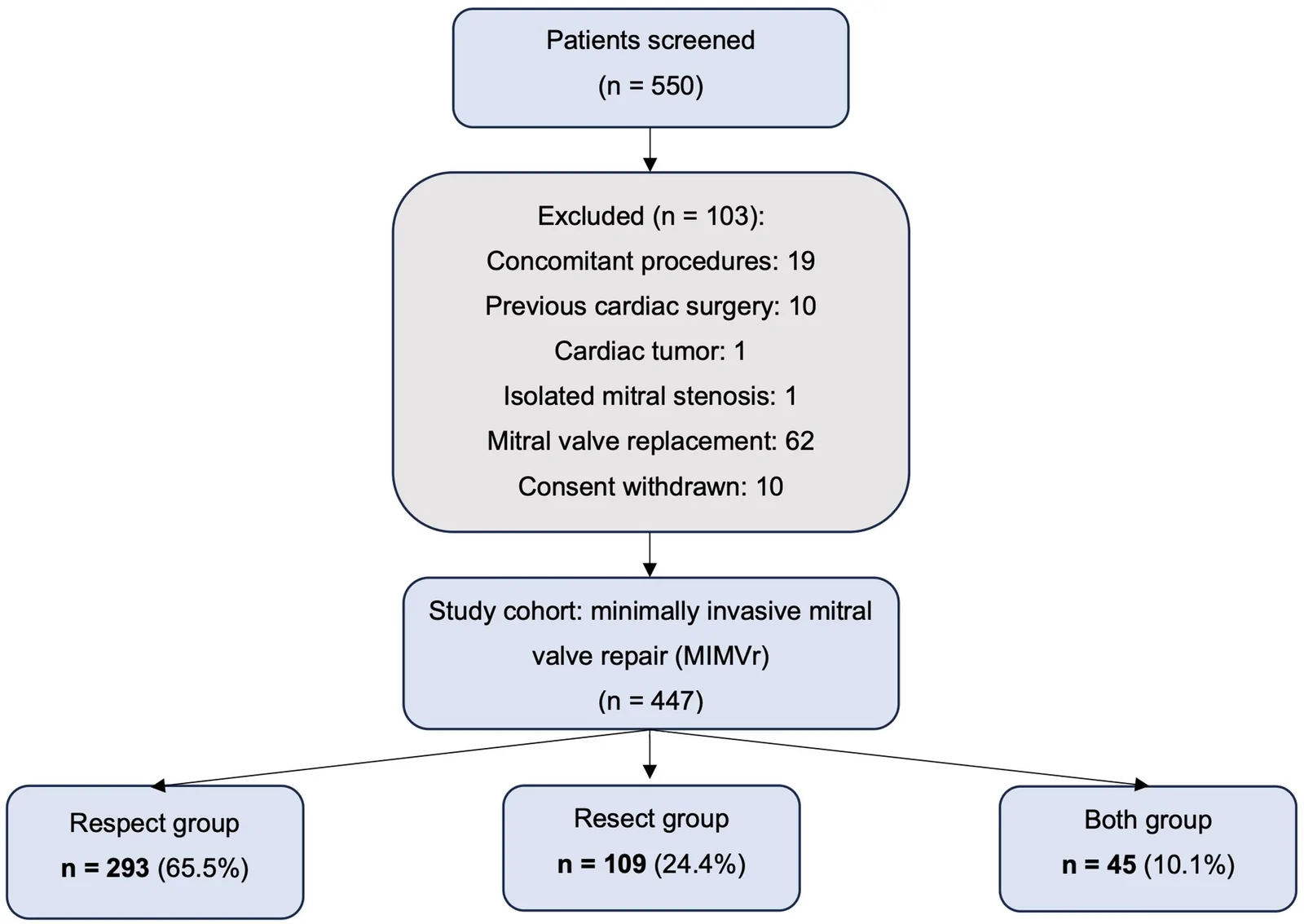
Flow chart illustrating patient selection and allocation to surgical treatment groups

Accordingly, 447 patients comprised the study cohort (Fig. 1).

Patients were stratified by repair strategy into ‘Respect’ (*n* = 293, 65.5%), ‘Resect’ (*n* = 109, 24.4%), and ‘Both’ (*n* = 45, 10.1%). Baseline characteristics are presented in Table 1. Mean EuroSCORE II was 2.6 ± 2.6 and differed significantly across repair strategies (*P* = 0.022), with the highest values observed in the ‘Resect’ group. Posterior mitral leaflet (PML) prolapse was the predominant pathology (87.9%) and occurred most frequently in the ‘Resect’ group (97.3%; *P* = 0.002). Barlow’s disease was present in 19.7% of patients and was significantly more common in the ‘Both’ group (48.9%; *P* < 0.001). NYHA class III or IV was present in 31.3% of patients.

**Table 1:** Baseline Characteristics

	MVr Total (*n* = 447)	Respect (*n* = 293)	Resect (*n* = 109)	Both (*n* = 45)	*P*
Age	60.7 ± 12.6	60.9 ± 12.7	61.5 ± 12.3	57.3 ± 13.1	0.168
Female	131 (29.3)	80 (27.3)	35 (32.1)	16 (35.6)	0.401
BMI	25.1 ± 3.7	25.2 ± 3.6	25.2 ± 3.8	24.0 ± 3.8	0.082
EuroSCORE II	2.6 ± 2.6	2.5 ± 2.7	2.8 ± 2.3	2.1 ± 1.8	**0.022**
Ejection fraction	61.4 ± 9.8	61.2 ± 10.7	61.6 ± 8.1	62.6 ± 7.7	0.676
NYHA 1	137 (30.6)	99 (33.8)	26 (23.9)	12 (26.7)	0.187
NYHA 2	170 (38.0)	99 (33.8)	50 (45.9)	21 (46.7)	
NYHA 3	119 (26.6)	82 (28.0)	26 (23.9)	11 (24.4)	
NYHA 4	21 (4.7)	13 (4.4)	7 (6.4)	1 (2.2)	
Barlow’s disease	88 (19.7)	47 (16.0)	19 (17.4)	22 (48.9)	**< 0.001**
Posterior leaflet prolapse	393 (87.9)	247 (84.3)	106 (97.3)	40 (88.9)	**0.002**
Atrial fibrillation	113 (25.3)	81 (27.7)	23 (21.1)	9 (20.0)	0.281
Hypertension	352 (78.7)	238 (81.2)	83 (76.2)	31 (68.9)	0.127
Diabetes mellitus	21 (4.7)	17 (5.8)	3 (2.8)	1 (2.2)	0.311
COPD	23 (5.1)	18 (6.1)	5 (4.6)	0 (0.0)	0.211
History of tobacco use	140 (31.3)	102 (34.8)	27 (24.8)	11 (24.4)	0.087

Values are presented as mean ± standard deviation or number (percentage). Bold values indicate statistically significant P-values (P ≤ 0.05).

### Operative parameters

Intraoperative variables are presented in Table 2. CPB and ACC times differed significantly between groups, being shortest in the ‘Respect’ group and longest in the ‘Resect’ group (*P* < 0.001). Among patients receiving an annuloplasty ring, mean ring size was 32.08 ± 3.03 mm in the ‘Respect’ group (*n* = 284), 32.79 ± 2.83 mm in the ‘Resect’ group (*n* = 107), and 34.31 ± 2.70 mm in the ‘Both’ group (*n* = 45), resulting in an overall mean of 32.48 ± 3.02 mm among ring recipients (*n* = 436; *P* < 0.001). Nadir core temperature during CPB was lower in the ‘Resect’ group (31.9 ± 2.3 °C vs. 33.6 ± 1.1 °C in ‘Respect’; *P* < 0.001). Neochordae were implanted in 79.1% of patients in the ‘Respect’ and in 97.8% of the ‘Both’ group, whereas none were used in the ‘Resect’ group (*P* < 0.001). Notably, 20.8% of patients in the ‘Respect’ group underwent isolated ring annuloplasty without concomitant leaflet intervention or neochordae implantation. Among these 61 isolated-annuloplasty patients, 27 had posterior leaflet prolapse without Barlow’s disease, 11 had Barlow’s disease, and 23 had other degenerative morphologies not meeting the criteria for posterior leaflet prolapse or Barlow’s disease. Left atrial cryoablation was performed in 19.0% of patients. No conversion to median sternotomy occurred in the MVr cohort.

**Table 2:** Intraoperative Parameters

	MVr Total (*n* = 447)	Respect (*n* = 293)	Resect (*n* = 109)	Both (*n* = 45)	*P*
Cardiopulmonary bypass (CPB) time (min)	142.7 ± 43.7	134.3 ± 40.3	161.6 ± 48.6	152.0 ± 36.1	< 0.001
Aortic cross-clamp time (min)	94.1 ± 30.5	87.5 ± 29.4	107.4 ± 29.4	104.9 ± 26.3	**< 0.001**
Nadir core temperature during CPB (°C)	33.2 ± 1.7	33.6 ± 1.1	31.9 ± 2.3	33.0 ± 1.9	**< 0.001**
Isolated annuloplasty ring	61 (13.6)	61 (20.8)	0 (0.0)	0 (0.0)	**< 0.001**
Annuloplasty ring size (mm)	32.48 ± 3.02	32.08 ± 3.03	32.79 ± 2.83	34.31 ± 2.70	**< 0.001**
Neochordae	275 (61.7)	231 (79.1)	0 (0.0)	44 (97.8)	**< 0.001**
Left atrial cryoablation	85 (19.0)	60 (20.5)	19 (17.4)	6 (13.3)	0.466
Left atrial appendage closure	27 (6.0)	21 (7.2)	3 (2.8)	3 (6.7)	0.251
PFO Closure	45 (10.1)	30 (10.2)	11 (10.1)	4 (8.9)	0.961

Values are presented as mean ± standard deviation or number (percentage). Annuloplasty ring size is reported among ring recipients: MVr Total *n* = 436, ‘Respect’ *n* = 284, ‘Resect’ *n* = 107, ‘Both’ *n* = 45. Bold values indicate statistically significant P-values (P ≤ 0.05).

### In-hospital outcomes

In-hospital outcomes are shown in Table 3. The mean intensive care unit (ICU) stay was 1.9 ± 2.5 days (median 1 [IQR 1–2]) and the mean total hospital stay was 10.4 ± 4.35 days (median 9 [IQR 8–12]). The overall in-hospital mortality was 0.7% (*n* = 3), and in-hospital stroke occurred in 0.9% (*n* = 4). The three in-hospital deaths were attributed to hypoxic encephalopathy following postoperative bleeding complications requiring repeated re-thoracotomy, with no documentation of cardiopulmonary bypass failure (postoperative day 3), multiorgan failure secondary to bronchopleural fistula and acute respiratory distress syndrome (ARDS) (postoperative day 10), and refractory hypotension followed by withdrawal of life-sustaining therapy at the patient’s request (postoperative day 9). Of the four cerebrovascular events, two were transient ischemic events (postoperative days 2 and 5), whereas two were permanent ischemic strokes with hemiparesis (postoperative days 2 and 3), confirmed by neuroimaging. In-hospital mitral valve–related reoperations occurred in 1.3% of patients (*n* = 6), with no significant intergroup difference. Indications included systolic anterior motion (*n* = 3; postoperative days 0, 2, and 9), annuloplasty ring dehiscence (*n* = 1; postoperative day 7), and recurrent relevant MR before discharge (*n* = 2; both on postoperative day 8). Re-exploration for bleeding occurred in 8.5%, reflecting a low institutional threshold aimed at minimizing transfusion requirements and preventing hemodynamic compromise. A surgical bleeding focus was documented in only three patients (one right ventricular bleeder, one from a right intercostal artery, and one from a small pericardial vessel). Third-degree atrioventricular block occurred in 1.3% (*n* = 6). Eight patients (1.8%) required a permanent pacemaker implantation with no significant group differences (*P* = 0.630).

**Table 3:** In-Hospital Outcomes

	MVr Total (*n* = 447)	Respect (*n* = 293)	Resect (*n* = 109)	Both (*n* = 45)	*P*
ICU stay (days)	1.9 ± 2.5	2.0 ± 2.8	1.7 ± 1.5	1.8 ± 1.9	0.579
Mortality	3 (0.7)	2 (0.7)	1 (0.9)	0 (0.0)	0.894
Stroke	4 (0.9)	2 (0.7)	2 (1.8)	0 (0.0)	0.440
Re-thoracotomy for bleeding	38 (8.5)	24 (8.2)	10 (9.2)	4 (8.9)	0.947
Pacemaker implantation	8 (1.8)	4 (1.4)	3 (2.8)	1 (2.2)	0.630
Groin cannulation site infection	4 (0.9)	4 (1.4)	0 (0.0)	0 (0.0)	0.346
Right groin lymph fistula / seroma	9 (2.0)	8 (2.7)	0 (0.0)	1 (2.2)	0.222
Postoperative cerebral edema	1 (0.2)	1 (0.3)	0 (0.0)	0 (0.0)	0.768
ECMO implantation	1 (0.2)	1 (0.3)	0 (0.0)	0 (0.0)	0.768
IABP insertion	1 (0.2)	0 (0.0)	1 (0.9)	0 (0.0)	0.211
In-Hospital mitral valve reoperation	6 (1.3)	3 (1.0)	1 (0.9)	2 (4.4)	0.162
Total hospital stay (days)	10.4 ± 4.35	10.5 ± 4.81	10.2 ± 3.17	10.2 ± 3.60	0.772

Values are presented as mean ± standard deviation or number (percentage). (ECMO = extracorporeal membrane oxygenation; IABP = intra-aortic balloon pump)

### Follow-up

Follow-up outcomes are summarized in Table 4. Patients with fewer than 30 days of follow-up were classified as lost to follow-up (*n* = 5), including two residing abroad, two untraceable after relocation, and one who emigrated without providing contact information. Mean follow-up duration was 60.0 ± 43.7 months and differed among groups (‘Respect’: 53.6 ± 41.4; ‘Resect’: 77.9 ± 46.1; ‘Both’: 57.7 ± 41.4 months; *P* < 0.001). Follow-up completeness increased from 68% at 1 year to 89% at 2 years due to additional data retrieval.

**Table 4:** Late Outcomes

	MVr Total (*n* = 447)	Respect (*n* = 293)	Resect (*n* = 109)	Both (*n* = 45)	*P*
Follow-Up (months)	60.0 ± 43.7	53.6 ± 41.4	77.9 ± 46.1	57.7 ± 41.4	< 0.001
Mitral valve–related reoperation	24 (5.4)	16 (5.5)	7 (6.4)	1 (2.2)	0.955
No MR	302 (67.6)	201 (68.6)	73 (67.0)	28 (62.2)	0.793
Mild MR	93 (20.8)	60 (20.5)	21 (19.3)	12 (26.7)	
Moderate MR	30 (6.7)	17 (5.8)	9 (8.2)	3 (6.7)	
Severe MR	22 (4.9)	15 (5.1)	6 (5.5)	2 (4.4)	
Mortality (all-cause)	26 (5.8)	15 (5.1)	8 (7.3)	3 (6.7)	0.673
Cardiac deaths	7 (1.6)	4 (1.4)	3 (2.7)	0 (0.0)	
Non-cardiac deaths	19 (4.2)	11 (3.7)	5 (4.6)	3 (6.7)	
MACCE	13 (2.9)	8 (2.7)	3 (2.7)	2 (4.4)	0.934
Stroke / TIA	11 (2.5)	7 (2.4)	2 (1.8)	2 (4.4)	
Myocardial infarction	2 (0.4)	1 (0.3)	1 (0.9)	0 (0.0)	
Incisional hernia	2 (0.4)	2 (0.7)	0 (0.0)	0 (0.0)	0.590

Values are presented as mean ± standard deviation or number (percentage). (MACCE = major adverse cardiac and cerebrovascular events)

**Fig. 2:**
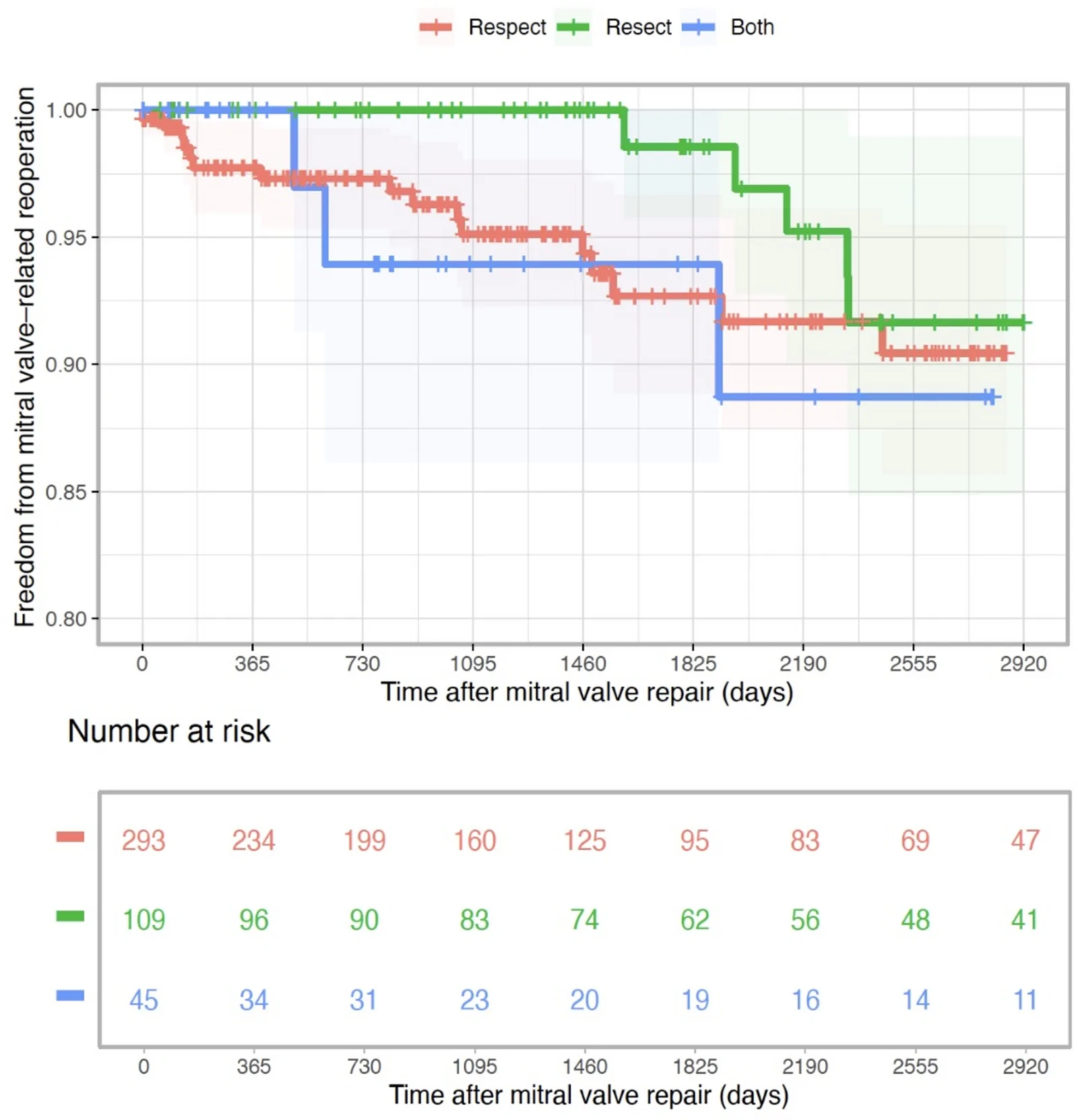
Freedom from mitral valve–related reoperation. Kaplan–Meier analysis illustrating freedom from mitral valve–related reoperation according to surgical technique. Five-year freedom from reoperation for the entire cohort was 93.3%. Event-free survival rates at 1, 2, and 5 years (95% CI) were as follows: ‘Respect’ 97.7% (95.9–99.5), 97.3% (95.4–99.3), 92.7% (88.9–96.7); ‘Resect’ 100.0% (100.0–100.0), 100.0% (100.0–100.0), 98.6% (95.8–100.0); ‘Both’ 100.0% (100.0–100.0), 93.9% (86.1–100.0), 93.9% (86.1–100.0). No statistically significant difference in freedom from reoperation was observed among groups (log-rank *P* = 0.647). Tick marks indicate censored observations

At follow-up, 88.4% of patients exhibited no or only mild MR, while moderate or severe recurrence was observed in 11.6%, with no significant intergroup differences (*P* = 0.793; Table 4). Kaplan–Meier estimates showed a 5-year freedom from mitral valve–related reoperation of 93.3% overall (92.7% ‘Respect’, 98.6% ‘Resect’, 93.9% ‘Both’; *P* = 0.647, log-rank; Fig. 2). During follow-up, 24 mitral valve–related reoperations (5.4%) were performed after discharge, with no significant differences between surgical strategies (*P* = 0.955; Table 4; Fig. 2).

Among the 24 late reoperations, indications included annuloplasty-ring dehiscence or partial detachment in 5 patients (20.8%), systolic anterior motion (SAM)–related obstruction of left ventricular outflow tract (LVOT) in 3 (12.5%), infective endocarditis in 1 (4.2%), and progressive leaflet prolapse or chordal failure in 5 (20.8%); the remaining 10 patients (41.7%) presented with mixed or complex mechanisms. The corresponding redo procedures among these 24 cases comprised redo mitral valve repair (re-MVr) in 9 patients (37.5%), mitral valve replacement with a bioprosthesis in 7 (29.2%), replacement with a mechanical prosthesis in 5 (20.8%), and transcatheter edge-to-edge repair (MitraClip™) in 3 (12.5%). In isolated cases, redo mitral valve procedures were combined with concomitant aortic valve replacement (*n* = 2) or coronary artery bypass grafting (*n* = 1).

**Fig. 3:**
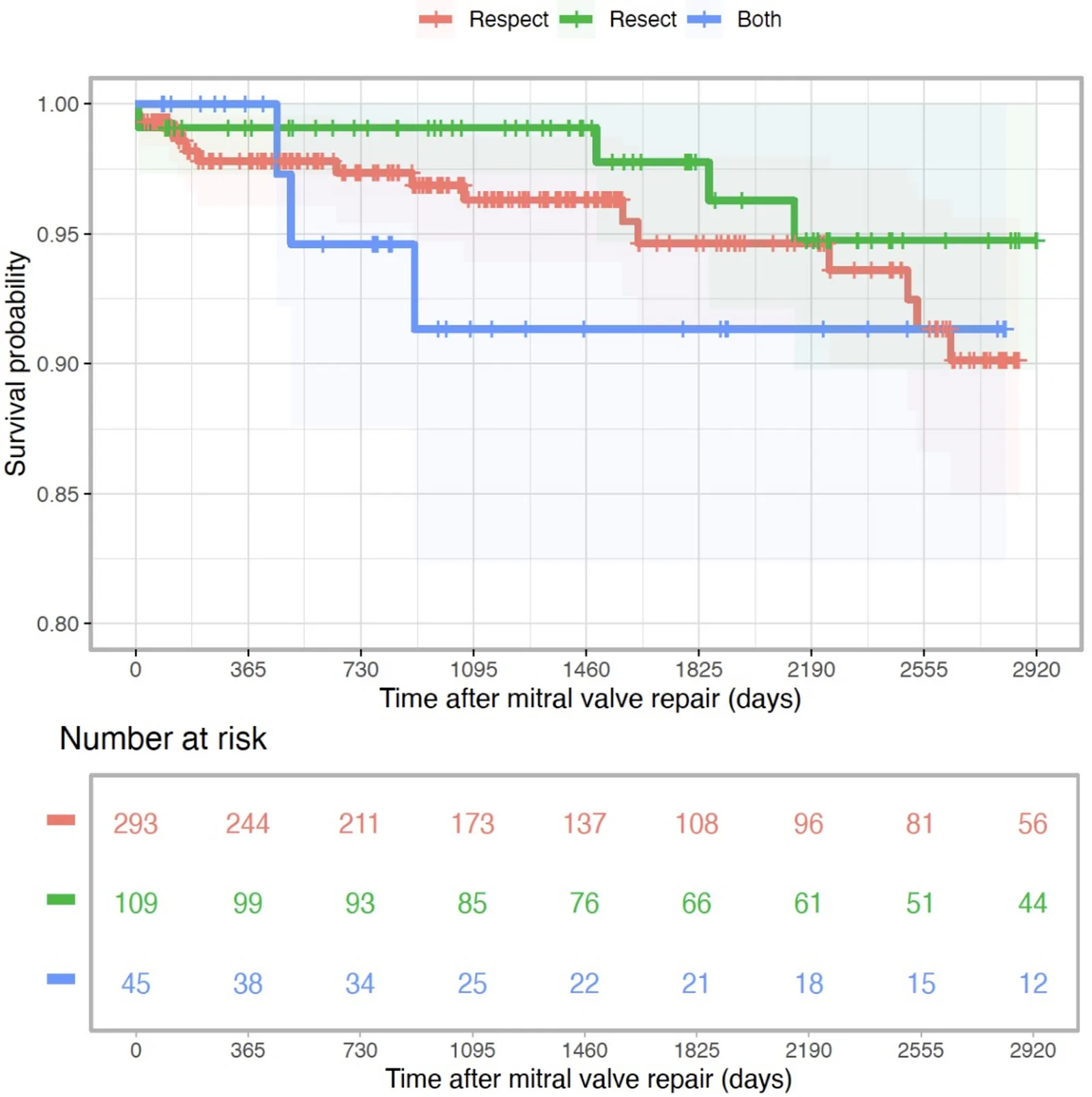
Overall survival. Kaplan–Meier analysis of overall survival according to surgical technique. Five-year survival for the entire cohort was 94.0%. Survival probabilities at 1, 2, and 5 years (95% CI) were as follows: ‘Respect’ 97.8% (96.1–99.6), 97.4% (95.4–99.3), 94.6% (91.4–98.0); ‘Resect’ 99.1% (97.3–100.0), 99.1% (97.3–100.0), 97.8% (94.7–100.0); ‘Both’ 100.0% (100.0–100.0), 94.6% (87.6–100.0), 91.3% (82.4–100.0). No statistically significant difference in overall survival was observed among groups (log-rank *P* = 0.964). Tick marks indicate censored observations

**Fig. 4:**
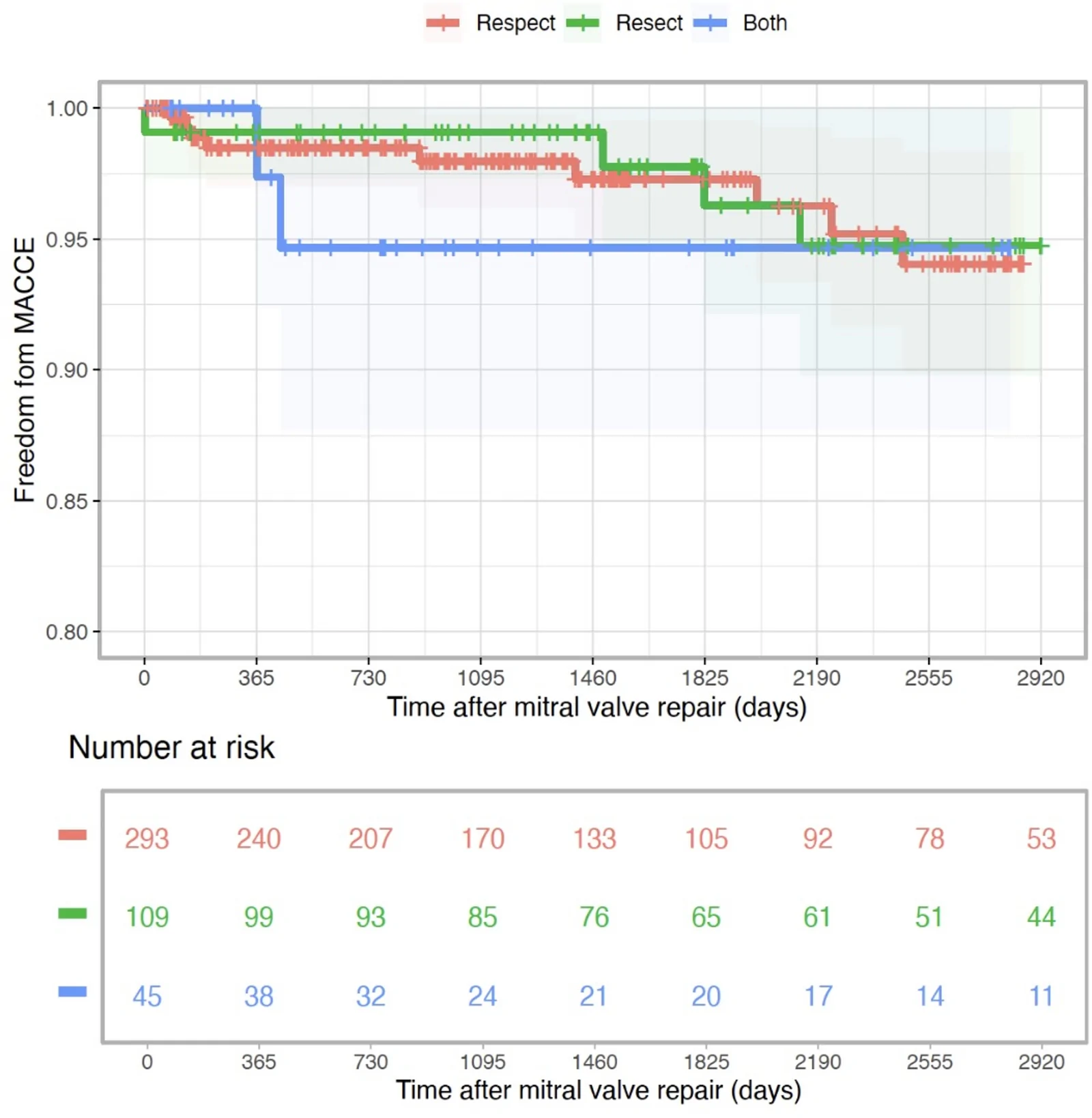
Freedom from major adverse cardiac and cerebrovascular events (MACCE). Kaplan–Meier analysis illustrating freedom from MACCE according to surgical technique. Five-year freedom from MACCE for the entire cohort was 96.8%. Event-free survival rates at 1, 2, and 5 years (95% CI) were as follows: ‘Respect’ 98.5% (97.0–100.0), 98.5% (97.0–100.0), 97.3% (95.1–99.5); ‘Resect’ 99.1% (97.3–100.0), 99.1% (97.3–100.0), 96.3% (92.2–100.0); ‘Both’ 100.0% (100.0–100.0), 94.7% (87.7–100.0), 94.7% (87.7–100.0). No statistically significant differences in MACCE-free survival were observed among groups (log-rank *P* = 0.896). Tick marks indicate censored observations

Overall 5-year survival was 94.0%, with no significant differences between repair strategies (*P* = 0.964; log-rank; Fig. 3). Five-year survival rates were 94.6% for the ‘Respect’ group, 97.8% for the ‘Resect’ group, and 91.3% for the ‘Both’ group (Fig. 3). During follow-up, 26 deaths occurred, including 19 non-cardiac and 7 cardiac deaths (Table 4). During follow-up, MACCE occurred in 2.9% of patients, predominantly driven by cerebrovascular events, whereas myocardial infarction was rare (Table 4). Five-year freedom from MACCE was 96.8% overall, with no significant differences among the ‘Respect’, ‘Resect’, and ‘Both’ groups (97.3%, 96.3%, and 94.7%, respectively; *P* = 0.896; log-rank; Fig. 4).

Incisional hernias occurred in 2 patients (0.4%), both involving the right mini-thoracotomy incision. Neither case required mesh repair. One patient presented with a right-sided thoracic scar hernia treated by direct fascial plication (primary suture repair), and another had a localized right chest wall hernia managed with primary wound closure without mesh.

### Competing risk analysis

In the competing risk model for mitral valve–related reoperation (death as the competing event), no significant differences were observed among surgical strategies. Using the ‘Respect’ group as the reference, the subdistribution hazard ratio (sHR) for the ‘Resect’ group was 0.50 (95% CI: 0.21–1.21; *P* = 0.120), and for the ‘Both’ group 1.42 (95% CI: 0.37–5.49; *P* = 0.610). No clinical or procedural variable was independently associated with the risk of reoperation.

Notably, no reoperations occurred among patients with COPD (*n* = 23), precluding estimation of a subdistribution hazard ratio; accordingly, COPD was omitted from the multivariable Fine–Gray model for reoperation and is reported as NE in Table 5.

As a secondary endpoint, MACCE was analyzed using a competing risk model with death as the competing event. No significant differences were observed among surgical strategies. Using the ‘Respect’ group as the reference, the sHR for the ‘Resect’ group was 1.31 (95% CI: 0.53–3.23; *P* = 0.550), and for the ‘Both’ group 2.24 (95% CI: 0.49–10.3; *P* = 0.300). None of the clinical covariates included in the multivariable model demonstrated a statistically significant association with MACCE.

**Fig. 5:**
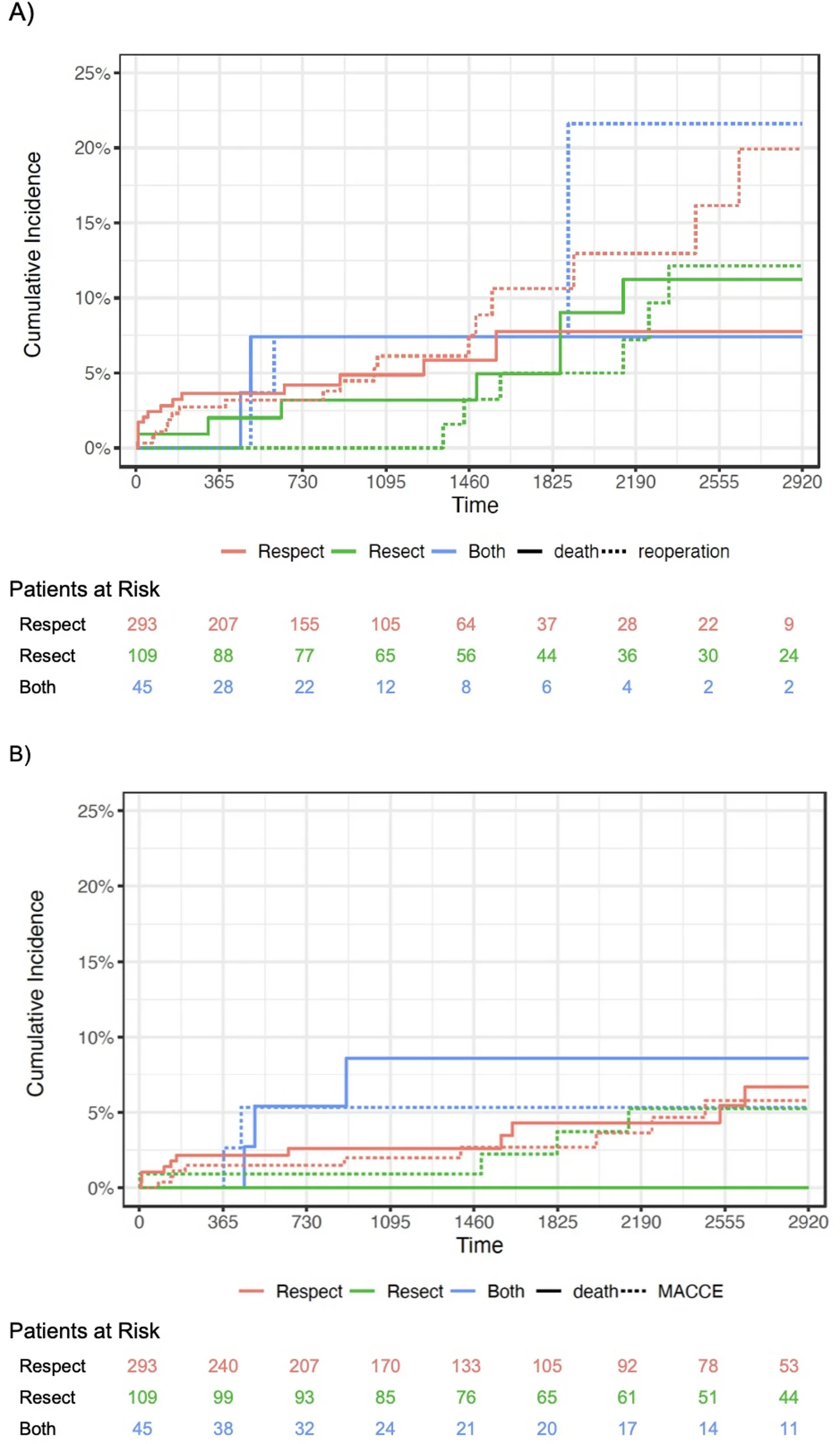
Competing risk analysis illustrating the cumulative incidence of **(A)** mitral valve–related reoperation and **(B)** major adverse cardiac and cerebrovascular events (MACCE), each with death as the competing event (Fine–Gray)

Cumulative incidence curves are displayed in Fig. 5A (mitral valve–related reoperation) and Fig. 5B (MACCE), each depicting the event-specific probability over time derived from the multivariable Fine–Gray subdistribution hazard model with death as the competing event; corresponding multivariable regression results for clinical covariates are provided in Tables 5 and 6. Variables not estimable due to zero events are denoted as NE.

**Table 5:** Competing Risk Regression for Reoperation (Death as Competing Risk)

Variable	sHR (95% CI)	*P*
Age (per year)	0.97 (0.94–1.00)	0.091
BMI	1.01 (0.91–1.13)	0.810
Atrial fibrillation	1.33 (0.50–3.53)	0.570
Ejection fraction	1.02 (0.97–1.07)	0.440
EuroSCORE II	1.07 (0.92–1.26)	0.380
Diabetes	1.97 (0.60–6.49)	0.260
COPD	NE (—)	—
ACC (min)	1.00 (0.97–1.02)	0.710
CPB Time (min)	1.01 (0.99–1.02)	0.330

sHR indicates subdistribution hazard ratio derived from Fine–Gray competing risk regression, with death treated as the competing event. Results are reported as sHR with 95% confidence intervals (CI). NE indicates not estimable due to zero events. Corresponding cumulative incidence curves are shown in Fig. 5A

**Table 6:** Competing Risk Regression for Major adverse cardiac and cerebrovascular events (MACCE) (Death as Competing Risk)

Variable	sHR (95% CI)	*P*
Age (per year)	1.00 (0.95–1.05)	0.990
BMI	0.95 (0.82–1.11)	0.530
Atrial fibrillation	2.58 (0.72–9.24)	0.150
Ejection fraction	0.99 (0.94–1.04)	0.710
EuroSCORE II	0.99 (0.82–1.19)	0.920
Diabetes	2.76 (0.70–10.90)	0.150
COPD	2.94 (0.57–15.20)	0.200
ACC (min)	0.98 (0.95–1.01)	0.230
CPB Time (min)	1.01 (0.99–1.03)	0.410

sHR indicates subdistribution hazard ratio derived from Fine–Gray competing risk regression, with death treated as the competing event. Results are reported as sHR with 95% confidence intervals (CI). Corresponding cumulative incidence curves are shown in Fig. 5B

## Discussion

In this single-center cohort of MIMVr, ‘Respect’, ‘Resect’, and ‘Both’ were associated with favorable early and longer-term outcomes, even among patients with advanced degenerative pathology. Despite longer ACC and CPB times in the ‘Resect’ and ‘Both’ groups, no differences were observed in in-hospital morbidity and mortality, longer-term survival, freedom from mitral valve–related reoperation, or MACCE, supporting a morphology-guided, patient-tailored strategy over rigid adherence to a single repair philosophy.

Most comparative studies of resectional versus non-resectional repair—particularly in isolated posterior leaflet prolapse—have focused on anatomically straightforward lesions, limiting extrapolation to complex degenerative phenotypes [22, 23]. By contrast, our findings support the feasibility of a minimally invasive approach even in complex degenerative disease, consistent with evidence demonstrating outcomes comparable to sternotomy [24–28] and aligned with the contemporary ‘less is Mohr’ philosophy of MIMVr, as originally proposed by Mohr et al. [29].

High-volume centers (including Leipzig) have demonstrated that morphology-guided repair via right mini-thoracotomy, combined with systematic annuloplasty and advanced reconstructive techniques, achieves high repair rates, durable valve competence, and low reoperation rates across the full spectrum of degenerative mitral valve disease [30–32]. This is supported by the multicenter analysis by Tomšič et al. in Barlow’s disease (*n* = 209), reporting low perioperative morbidity and no relevant differences in early mortality between resectional and non-resectional strategies [33].

At midterm follow-up, Tomšič et al. demonstrated sustained durability (≈ 96% survival at six years; ≈98–99% freedom from reintervention), irrespective of the repair strategy applied [33]. In our broader real-world MIMVr cohort, outcomes were concordant, with a five-year survival of 94.0% and a five-year freedom from mitral valve–related reoperation of 93.3%. Together, these data indicate that morphology-guided repair in experienced centers is associated with low perioperative risk and durable midterm outcomes across complex degenerative pathology.

A meta-analysis by Mazine et al. and a prospective comparative study by Imasaka et al. reported superior preservation of left ventricular function following neochordal repair for PML prolapse, supporting the ‘Respect’ technique as a more physiologic approach in selected anatomies and consistent with preservation of subvalvular geometry [22, 23].

Multicenter cohorts, registry analyses, and long-term MIMVS series corroborate the safety and durability of MIMVr across heterogeneous real-world populations; contemporary comparative studies likewise report equivalent early and midterm outcomes among resectional, neochordal, and hybrid strategies when applied in a morphology-tailored manner [18, 28, 34–37].

Collectively, these convergent data provide a robust external framework within which the findings of the present cohort integrate, reinforcing the principle that long-term success in degenerative mitral valve surgery is governed primarily by morphology-guided repair rather than adherence to a singular operative doctrine. In this context, our real-world experience demonstrates the safety, efficacy, and durability of contemporary MVr and affirms its role as a benchmark for emerging reconstructive strategies.

Several recent studies confirm that MIMVr does not compromise repair quality or durability. This is consistent with the conceptual work by Holubec et al., who highlighted that both resectional and neochordal techniques can be applied safely in the minimally invasive setting and that chordal-preserving (‘Respect’) strategies may confer physiological advantages when appropriately indicated [13]. Subsequent contributions by Holubec et al. emphasized that the maturation of minimally invasive mitral repair is driven by structured team expertise and a disciplined progression toward endoscopic visualization, thereby reinforcing the conceptual foundations of contemporary MIMVS [38]. In a comparable real-world MIMVr cohort, Passos et al. reported high procedural success (94.5%) and durable midterm outcomes, with a 5-year freedom from reoperation of 96.5%, reflecting results achieved beyond the initial learning curve [26, 34, 36]. Collectively, these data underscore that surgical expertise and technical execution—rather than access route per se—constitute the principal determinants of outcome, yielding consistent results across patient subgroups within structured heart-team pathways [39, 40].

Concordant with this concept, our cohort—despite substantial morphological heterogeneity and the deliberate inclusion of early learning-curve cases—demonstrated favorable perioperative outcomes, including low in-hospital mortality (0.7%) and stroke incidence (0.9%). Access-related morbidity was minimal, with only two incisional hernias (0.4%) and no mini-thoracotomy-related wound infections, underscoring the favorable wound profile of the right mini-thoracotomy approach. Sustained mid-term valve competence was achieved, in keeping with contemporary high-volume MIMVS experience, even in complex phenotypes such as Barlow’s disease, bileaflet prolapse, and anterior leaflet involvement [16, 17, 26, 27, 30–32, 36].

With respect to long-term durability, the present analysis deliberately focused on sustained valve competence over extended follow-up. This approach aligns with mature long-term MIMVr experience reported in the literature, including recent evaluations by Salem et al., which situate right-sided minimally invasive approaches within a broader continuum of durable reconstructive strategies [41]. Accordingly, the cohort was restricted to procedures performed between 2006 and 2014, enabling extended follow-up (maximum 15 years and 9 months) and confirming durable repair outcomes, with mitral regurgitation ≤grade I in 88.4% of patients and a low in-hospital reintervention rate of 1.3%.

By analyzing a real-world MIMVr cohort characterized by substantial anatomical complexity, we demonstrate uniformly favorable longer-term outcomes, with sustained valve competence and low rates of MACCE and reoperation. These findings reinforce the growing consensus that durable success in mitral valve repair is best achieved through pathology-driven, morphology-tailored strategies rather than adherence to a uniform, one-size-fits-all paradigm.

The absence of significant clinical predictors in the competing risk models most likely reflects the very low MACCE event rate in this selected degenerative MVr cohort, leading to limited statistical power and wide confidence intervals; accordingly, covariate effects should be interpreted cautiously.

The ‘Respect’ group (65.5%) demonstrated significantly shorter CPB and ACC times than ‘Resect’ and ‘Both’ (CPB 134 ± 40 vs. 162 ± 49 and 152 ± 36 min; *P* < 0.001), consistent with lower procedural complexity and/or greater technical efficiency. Prior work by Del Forno et al. similarly reported reduced operative times with comparable procedural safety for chordal-based repair in predominantly straightforward PML prolapse [18]. Broader MIMVr cohorts likewise emphasize that outcomes are primarily driven by surgical expertise and morphology-guided technique rather than strict adherence to a single strategy [34, 42]. By contrast, the ‘Resect’ group exhibited a significantly higher prevalence of isolated PML prolapse (97.3%), whereas ‘Both’ was enriched for anatomically complex cases such as Barlow’s disease (48.9%). This distribution aligns with prior literature emphasizing the utility of hybrid techniques in Barlow’s morphology, where excessive leaflet tissue and bileaflet involvement often preclude pure ‘Resect’ or ‘Respect’ strategies [27, 31]. Sakaguchi et al. demonstrated that Barlow’s disease can be systematically addressed via a stepwise minimally invasive repair concept, supporting the use of hybrid techniques in anatomically complex valves [27]. Despite these anatomical and procedural distinctions, early outcomes remained uniformly favorable, underscoring the adaptability of MIMVr even in anatomically complex degenerative lesions such as Barlow’s disease, extensive bileaflet prolapse, multisegment posterior or anterior leaflet prolapse, and commissural involvement.

### Study strengths and limitations

This study reports a large consecutive cohort of patients undergoing minimally invasive mitral valve repair, with structured prospective follow-up and clinically meaningful endpoints, including recurrent mitral regurgitation, mitral valve–related reoperation, survival, and MACCE. By deliberately encompassing anatomically complex degenerative phenotypes—such as Barlow’s disease, bileaflet pathology, and anterior leaflet involvement—the analysis extends comparative evidence beyond isolated posterior leaflet prolapse. The application of competing risk regression strengthens inference for reoperation and MACCE by appropriately accounting for death as a competing event and yielding clinically interpretable cumulative incidence estimates. Importantly, cases requiring conversion to mitral valve replacement after an initially intended repair, reflecting the failed-repair pathway, were systematically captured and reported separately, thereby preserving methodological transparency and preventing conflation of repair- and replacement-related trajectories. Finally, the favorable early safety profile and minimal access-related morbidity underscore the clinical applicability of a morphology-guided, patient-tailored strategy within a minimally invasive setting.

Several limitations merit consideration when interpreting the present findings. First, the retrospective, single-center design inherently introduces the potential for selection bias and limits the generalizability. Although the cohort reflects real-world practice across a broad spectrum of degenerative mitral valve pathology, patient selection, surgical decision-making, and perioperative management were influenced by institutional expertise and may not be reproducible in lower-volume or less specialized centers.

Second, patients undergoing intraoperative conversion from an initially intended repair to mitral valve replacement, as well as those in whom replacement was performed de novo after direct intraoperative assessment, were excluded from the primary analyses. While this approach may introduce selection bias, it was predefined to ensure a homogeneous cohort with a successfully reconstructed native mitral valve and to allow a focused evaluation of repair-specific outcomes. These cases are presented separately to maintain transparency while preventing dilution of the repair cohort by fundamentally different clinical trajectories.

Third, allocation to surgical strategy—‘Respect’, ‘Resect’, or ‘Both’—was determined intraoperatively based on valve morphology and surgeon judgment and experience rather than randomization. Consequently, comparisons between groups remain susceptible to confounding by indication. While baseline characteristics were broadly balanced, certain morphological features—such as Barlow’s disease or anterior leaflet involvement—were more prevalent in certain groups and may have influenced outcomes.

Fourth, echocardiographic follow-up was not adjudicated by an independent core laboratory. While imaging was prospectively scheduled and largely complete, interobserver variability across centers and individual cardiologists cannot be excluded and may have affected the grading of residual or recurrent mitral regurgitation.

Finally, despite a maximum follow-up exceeding 15 years, median follow-up was approximately five years; late events—including reintervention or structural deterioration—may be underrepresented, and the relatively small number of patients reaching long-term endpoints such as MACCE or reoperation may have limited statistical power to detect subtle differences between repair strategies.

## Conclusion

In this single-center cohort of MIMVr for DMR, all three surgical strategies—‘Respect’, ‘Resect’, and ‘Both’—demonstrated comparable safety and longer-term durability even among anatomically complex degenerative valve pathologies. Despite inclusion of the procedural learning curve and longer cross-clamp times in the ‘Resect’ and ‘Both’ groups, in-hospital outcomes remained comparable. At follow-up, no significant differences were observed in freedom from mitral valve–related reoperation, nor in overall survival or MACCE. Competing risk regression confirmed the absence of intergroup differences. Our results support a morphology-guided, patient-tailored surgical approach as a reliable and durable standard for DMR within a minimally invasive setting.

## Data Availability

The datasets generated and/or analyzed during the current study are not publicly available due to patient privacy and institutional regulations but are available from the corresponding author on reasonable request.

## Abbreviations

ACC: Aortic cross-clamp (time)
AF: Atrial fibrillation
ARDS: Acute respiratory distress syndrome
BMI: Body mass index
CI: Confidence interval
COPD: Chronic obstructive pulmonary disease
CPB: Cardiopulmonary bypass (time)
DMR: Degenerative mitral regurgitation
ECMO: Extracorporeal membrane oxygenation
ESC: European Society of Cardiology
EACTS: European Association for Cardio-Thoracic Surgery
ICU: Intensive care unit
IABP: Intra-aortic balloon pump
IQR: Interquartile range
KEK: Cantonal Ethics Committee Zurich (Kantonale Ethikkommission Zürich)
LAA: Left atrial appendage
LVEF: Left ventricular ejection fraction
LV: Left ventricle/left ventricular
LVOT: Left ventricular outflow tract
MACCE: Major adverse cardiac and cerebrovascular events
MIMVS: Minimally invasive mitral valve surgery
MIMVr: Minimally invasive mitral valve repair
MR: Mitral regurgitation
MV: Mitral valve
MVr: Mitral valve repair
MVR: Mitral valve replacement
NYHA: New York Heart Association (functional class)
PFO: Patent foramen ovale
PML: Posterior mitral leaflet
R: R Foundation for Statistical Computing (software environment)
SAM: Systolic anterior motion
SD: Standard deviation
sHR: Subdistribution hazard ratio
SPSS: Statistical Package for the Social Sciences (software)
TEE: Transesophageal echocardiography
TIA: Transient ischemic attack
